# The Long-Term Outcome Comparison of Different Time-Delayed Kallikrein Treatments in a Mouse Cerebral Ischemic Model

**DOI:** 10.1155/2018/1706982

**Published:** 2018-04-05

**Authors:** Yaohui Ni, Kefu Cai, Yujie Hu, Chuan-hui Wang, Yuanyuan Zhang, Hua Huang, Xing Su, Jin-hua Gu

**Affiliations:** ^1^Department of Neurology, Affiliated Hospital of Nantong University, Jiangsu 226001, China; ^2^Department of Pathophysiology, Medical School of Nantong University, Jiangsu 26001, China; ^3^Geriatric Department, Shanghai Ninth People's Hospital, Shanghai Jiaotong University School of Medicine, Shanghai 201900, China; ^4^Department of Pathology, Affiliated Hospital of Nantong University, Jiangsu 226001, China; ^5^Department of Neurosurgery, Affiliated Hospital of Nantong University, Jiangsu 226001, China

## Abstract

Delayed administration of kallikrein after cerebral infarction can improve neurological function. However, the appropriate kallkrein treatment time after ischemic stroke has not been illuminated. In this study, we compared the long-term outcome among three kallikrein therapeutic regimens starting at different time points following mouse cerebral ischemia. Furthermore, the protective mechanisms involving neurogenesis, angiogenesis, and AKT-GSK3*β*-VEGF signaling pathway were analyzed. Human tissue kallikrein was injected through the tail vein daily starting at 8 h, 24 h, or 36 h after right middle cerebral artery occlusion (MCAO) until the 28th day. Three therapeutic regimens all protected against neurological dysfunction, but kallikrein treatment starting at 8 h after MCAO had the best efficacy. Additionally, kallikrein treatment at 8 h after MCAO significantly enhanced cell proliferation including neural stem cell and induced differentiation of neural stem cell into mature neuron. Kallikrein treatment starting at 8 h also promoted more angiogenesis than other two treatment regimens, which was associated with AKT-GSK3*β*-VEGF signaling pathway. Thus, we confirm that three delayed kallikrein treatments provide protection against cerebral infarction and furthermore suggest that kallikrein treatment starting at 8 h had a better effect than that at 24 h and 36 h. These findings provide the experimental data contributing to better clinical application of exogenous kallikrein.

## 1. Introduction

Stroke is the leading cause of death and disability worldwide [1]. Like other countries, ischemic stroke is the most common type of stroke in China [2]. According to the guidelines from the American Stroke Association for the early management of patients with acute cerebral ischemia, intravenous administration of rtPA remains the only recommended pharmacological therapy within 4.5 hours after acute ischemic stroke [3]. More than 4.5 hours after acute ischemic stroke, therapies are being sought to improve functional neurological recovery and reduce disability.

As an important element of the kallikrein/kinin system (KKS), tissue kallikrein can cleave low-molecule kininogen into kinins (e.g., bradykinin and kallidin). The biological function of tissue kallikrein is mainly produced by kinin binding to high-affinity kinin B1 or B2 receptors [4]. The B2 receptor is constitutively expressed, whereas the B1 receptor is expressed at very low levels under normal conditions and is triggered by inflammation or stress. Intact kinins can bind to the kinin B2 receptor, and the metabolites of kininase I, such as des-Arg9-BK and des-Arg10-kallidin, bind to the kinin B1 receptor. Additionally, kallikrein can also activate the B2 receptor directly. All components of the KKS are expressed in the brain. After ischemic stroke, the KKS is activated and the expression of kinin, kinin B1, and kinin B2 receptors is upregulated [5–7]. Human tissue kallikrein gene transfer immediately after ischemia-reperfusion injury provides neuroprotection against cerebral ischemia injury by the kinin B2 receptor in the rat MCAO model [8]. Moreover, delayed kallikrein administration by systemic gene delivery at 8 h after MCAO was also effective in reducing neurological deficit scores and cerebral infarction without affecting blood pressure [9]. Similarly, delayed kallikrein protein administration at 24 h after focal brain infarction significantly reduced neurological deficits in hypertensive rats [10]. Therefore, compared with tPA, tissue kallikrein has a beneficial effect in experimental murine stroke model with a wide time window of several days. More importantly, in a multicenter and double-blind clinical trial, human urinary kallidinogenase (HUK) was also effective when treated within 48 h after inpatients with stroke onset [11]. The data suggest that tissue kallikrein therapy is a promising treatment for acute human ischemic stroke. Based on more than 3000 cases of phase IV clinical studies in China, kallikrein therapy efficacy reached 88%, if patients were treated with human urinary kallikrein within 2 days. Thus, HUK has been approved by the Chines FDA as a novel drug for stroke patients.

However, some detailed information about delayed kallikrein treatment has not been explored, such as, whether different time point treatments after ischemia will cause efficacy difference and if so, which time point for the delayed kallikrein treatment will produce better protection. Thus, in the present study, we compared the effects of delayed kallikrein treatment starting at 8 h, 24 h, and 36 h after stroke onset in a cerebral ischemic mouse model. In addition, from the point of view of neurogenesis and angiogenesis, the underlying mechanism of effect difference was explored. The study provided the experimental data contributing to better clinical application of exogenous kallikrein.

## 2. Materials and Methods

### 2.1. Animal Middle Cerebral Artery Occlusion (MCAO) Surgery

Animal experiments were performed in accordance with the ARRIVE guidelines. Procedure for the use of laboratory animals was approved by the Institutional Animal Care and Use Committee of Nantong University, Jiangsu, China. During the animal studies, guidelines of *the regulation for the administration of affairs concerning experimental animals of China* enacted in 1988 were followed. A total of 95 male ICR mice weighing 25–30 g were used for this study. The MCAO surgery was performed as described previously. Briefly, mice were anesthetized with intraperitoneal injection of 2.5% Avertin. Through a ventral midline incision, the right common carotid artery, internal carotid artery, and external carotid artery were surgically exposed. A 6–0 nylon suture with silicon coating (Doccol Corporation, Redlands, CA) was inserted into the internal carotid artery through the external carotid artery stump and was gently advanced to occlude the middle cerebral artery. To obtain blood reperfusion, the occluding filament was withdrawn after occlusion for 1.5 hrs. Body temperature was maintained between 37.0°C and 37.5°C with a heating pad during surgery. Cerebral blood flow was monitored by Laser-Doppler flowmetry, and only those mice with 90% of blood flow blockade during MCAO and 85–95% recovery of blood flow during reperfusion were used for further experiments. The sham-operated mice underwent identical surgery, but the suture was not inserted. Mice that died within 6 hrs after cerebral artery occlusion procedure were excluded from the study. All experiments were performed in a randomized manner. Evaluation of neurological deficits and brain loss was performed by an investigator blinded to the experimental treatments.

### 2.2. Experimental Groups and Human Tissue Kallikrein and BrdU Labeling

Male ICR mice (*n* = 95) weighing 25–30 g were divided into five groups: group 1, the sham-operated mice (sham, *n* = 15). In a total of 80 MCAO mice, five mice died within 6 h after reperfusion. The rest MCAO mice were randomly assigned to 4 groups at 8 h after reperfusion: group 2, the mice suffered from MCAO alone without kallikrein treatment (MACO, *n* = 18); group 3, the mice suffered from MCAO plus kallikrein treatment daily for 28 consecutive days starting at 8 h after reperfusion (KLK 8 h, *n* = 18); group 4, the mice suffered from MCAO plus kallikrein treatment daily for 27 consecutive days starting at 24 h after reperfusion (KLK 24 h, *n* = 18); group 5, the mice suffered from MCAO plus kallikrein treatment daily for 27 consecutive days starting at 36 h after reperfusion (KLK 36 h, *n* = 19). The tissue kallikrein (Techpool Bio-Pharma Co. Ltd. Guangdong, China) was dissolved with normal saline. The mice in groups 3, 4, and 5 received a bolus injection of kellikrein daily through a tail vein at the dosage of 2.4 × 10^−2^ PNAU/kg. BrdU, which can incorporate into the DNA of dividing cells during S-phase, was used to label proliferative cells. BrdU (75 mg/kg, Sigma-Aldrich) was injected intraperitoneally twice daily for 5 consecutive days starting 48 h after reperfusion in all mice.

### 2.3. Neurological Functional Assessment and Brain Area Loss Measurement

In all animals, a battery of behavioral tests was performed at 14 and 28 days after MCAO by an investigator blinded to the experimental treatments. To evaluate neurological function, modified neurological severity score (mNSS) was applied [12].The mNSS contains the motor, sensory, balance, and reflex tests. Neurological function was graded on a scale of 0 to 18(normal score, 0; maximal score, 18).A higher score indicates a more severe injury. Additionally, pole test was used for evaluating the mouse movement disorder. The pole test was adapted to Matsuura et al. with minor modifications [13]. In brief, the mouse was placed head upward near the top of the pole, which was covered with a tape to create a rough surface. The time taken to turn completely downwards (T/turn) and the total time to reach the floor with all four paws (T/floor) were recorded. If the animal was unable to turn completely, the time to reach the floor was also attributed to T/turn. Each animal was tested on 5 trials, and the average score was taken as the final pole test score.

After removing and photographing the intact brains at 28 days after MCAO, brain area loss was determined using cresyl violet staining to assess the remaining area by four 20 *μ*m coronal sections cut on a cryostat. Sections were mounted on gelatin-coated microscope slides, incubated in 1.0% cresyl violet acetate (Sigma, St. Louis, MO) for 8 min then dehydrated sequentially in 70%, 95%, and 100% ethanol and xylene baths at room temperature. Percent brain loss was calculated as the area of the contralateral hemisphere minus the ipsilateral hemisphere/whole brain section × 100 [14].

### 2.4. Histology and Immunofluorescence and Cell Counting

Mice were deeply anesthetized, cardially perfused with normal saline, and decapitated, and brains were snap frozen in liquid nitrogen for cryostat sectioning at 14 days and 28 days after MCAO. Cryosections (20 *μ*m) were fixed with acetone/methanol for 10 min at −20°C and washed 3 times for 5 min with PBS. For BrdU double staining, sections were incubated with 2 N HCl for 30 min at 37°C, followed by an incubation in 0.1 M borate buffer for 10 min at room temperature, washed 6 times for 10 min with TBS, blocked in TBS with 10% normal goat serum and 0.1% Triton for 30 min, incubated with primary antibody against BrdU (1 : 800, Sigma-Aldrich), rabbit anti-Nestin (1 : 100, Sigma-Aldrich), mouse anti-NeuN (1 : 500, Chemicon), and mouse anti-Tuj1 (1 : 50, Abcam) at 4°C overnight in TBS with 1% normal goat serum and 0.1% Triton overnight, washed 2 times for 5 min with TBS and 1 time for 15 min with TBS with 1% normal goat serum and 0.1% Triton, incubated with secondary antibody (1 : 400) for 2 h washed 2 times for 5 min with PBS, incubated 3 min with DAPI, and washed 2 times with water. Sections were mounted and visualized in a confocal microscope (Leica), and photomicrographs were taken for further analysis. For the negative staining of NeuN and Tuj1, the sections were incubated without the primary antibody.

The quantification of antigen-positive cells was performed in six 10 *μ*m coronal sections per animal, spaced 200 *μ*m apart (4 fields per mouse; 4–6 mice per group). Cells were counted under high power (40 objective) on a microscope (Olympus IX51, Japan). Data were represented as a number of positive cells/mm^2^ of BrdU or double-positive cells per section. All quantifications were performed with the ImageJ image analysis software.

### 2.5. Western Blot Analyses

The ipsilateral cortex tissue of the mouse brain was homogenized in prechilled buffer containing 50 mM Tris-HCl (pH 7.4), 2.0 mM EGTA, 2 mM Na3VO4, 50 mM NaF, 0.5 mM AEBSF, 10 *μ*g/ml aprotinin, 10 *μ*g/ml leupeptin, and 4 μg/ml pepstatin A. Protein concentrations of the homogenates were determined by using Pierce 660 nm Protein Assay kit (Thermo Fisher Scientific Inc.). The samples were resolved in 10% sodium dodecyl sulfate (SDS)-polyacrylamide gel electrophoresis (PAGE) and electrotransferred onto PVDF membrane (Millipore, Bedford, MA). After blocking with 5% fat-free milk, the blots were then probed with a primary antibody, such as VEGF (1 : 1000; Abcam, USA), anti-AKT (1 : 1000; Cell Signaling Technology, USA), anti-pSer473-AKT (1 : 1000; Cell Signaling Technology, USA), anti-pSer9-GSK3*β* (1 : 1000; Cell Signaling Technology, USA), anti-GSK3*β* (1 : 1000; Cell Signaling Technology, USA), or anti-GAPDH (1 : 2000; Sigma, USA),washed and then incubated with a corresponding HRP-conjugated secondary antibody. The protein-antibody complex was visualized by using the Pierce ECL Western Blotting Substrate (Thermo Scientific) and exposed to a HyBlot CL autoradiography film (Denville Scientific, Inc. Metuchen, NJ). Specific immunostaining was quantified by using the Multi Gauge software V3.0 (Fuji Photo Film Co. Ltd.).

### 2.6. Statistical Analysis

The data were analyzed by one-way ANOVA followed by Tukey's post hoc tests or unpaired two-tailed *t* test using software Graphpad Prism 5. Numerical data were presented as means ± SD, and *p* < 0.05 was considered statistically significant.

## 3. Results

### 3.1. Long-Term Outcome of Delayed Kallikrein Treatment at Different Time Points in Mouse Cerebral Ischemic Models

**Figure 1:**
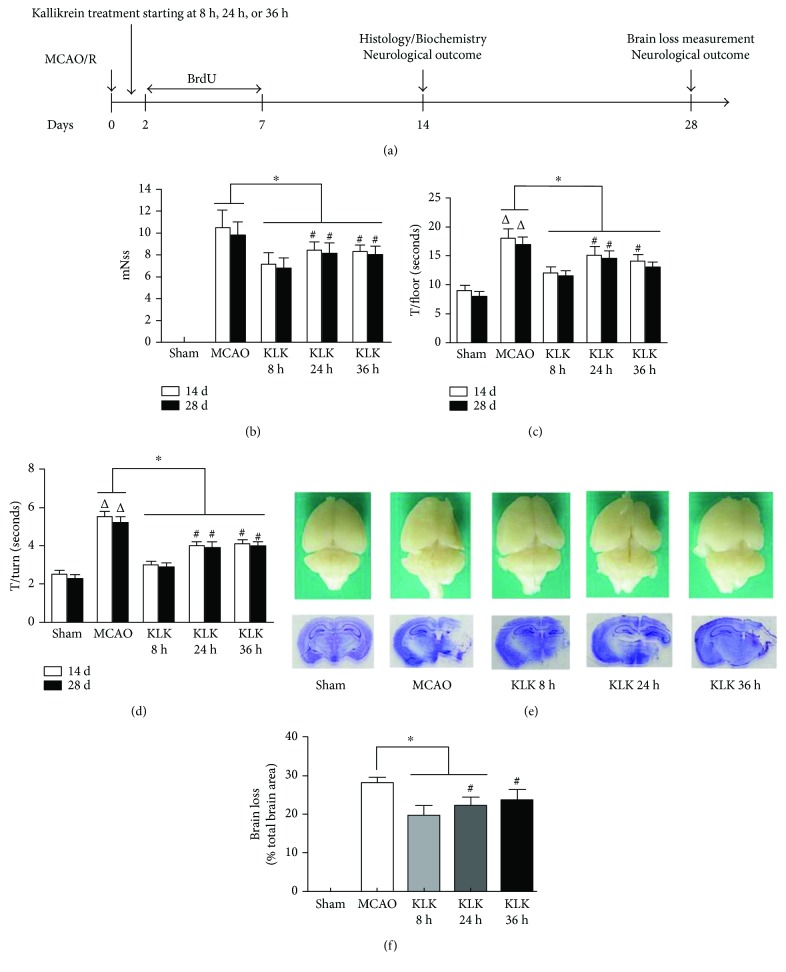
The effect of delayed kallikrein treatments on long-term outcome of brain ischemia. (a) Schematic presentation of the experimental design. (b, c, d) Neurological function (b) evaluated with mNSS and movement function (c, d) assessed by pole test at 14 d and 28 d after reperfusion injury. (e, f) Brain loss measurement at 28 d after reperfusion injury. Representative picture of intact brain and brain section stained by cresyl violet (e). Quantitative data of brain loss (f). ^∗^*p* < .05 compared with the MCAO group; ^△^*p* < 0.05 compared with the sham group; ^#^*p* < 0.05 compares with the KLK 8 h group.

To compare the efficacy of kallikrein starting at different time points, the neurological function of mice was assessed at 14 d and 28 d after MCAO/reperfusion injury (Figure 1(a)). We found all exogenous tissue kallikrein treatments starting at 8 h, 24 h, and 36 h (i.e., KLK 8 h, KLK 24 h, and KLK 36 h) after ischemic stroke can ameliorate the neurological deficits, but the KLK 8 h group has lower scores (better neurological outcome) than the KLK 24 h and KLK 36 h groups (Figures 1(b)–1(d)). There was no significant difference in neurological severity score between KLK 24 h and KLK 36 h groups.

After removing and photographing the whole brains of some mice in different groups at 28 d after MCAO, brain loss was analyzed by using cresyl violet staining (Figure 1(e)). The results showed that the brain loss areas in the KLK 8 h and KLK 24 h groups but not in the KLK 36 h group were significantly reduced compared with the MCAO group. Consistent with the neurological function, the brain loss area in the KLK 8 h group is much more smaller than that in the KLK 24 h and KLK 36 h groups (Figures 1(e) and 1(f)). These data indicate that though three kallikrein therapeutic regimens all can attenuate the neurological deficits in cerebral ischemic mouse model, kallikrein treatment starting at 8 h has the best efficacy.

### 3.2. Kallikrein Treatment Starting at 8 h after Ischemia Enhances More Cell Proliferation in the Peri-Infarction Area and the Ipsilateral SVZ

**Figure 2:**
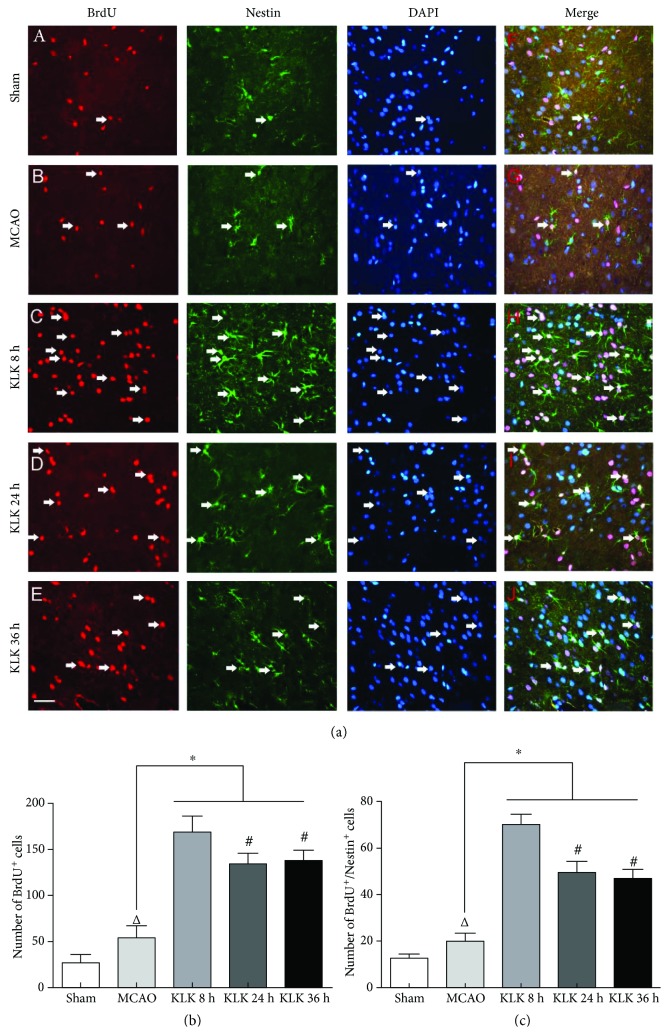
Administration of exogenous tissue kallikrein starting at 8 h, 24 h, and 36 h after ischemic stroke can increase BrdU^+^ cells (a, C–E) and BrdU^+^/Nestin^+^ cells (a, H–J) in the ipsilateral SVZ. Quantitative data of BrdU^+^ cells (b) and BrdU^+^/Nestin^+^cells (c) in the ipsilateral SVZ. ^∗^*p* < 0.05 compared with the MCAO group. ^△^*p* < 0.05 compared with the sham group. ^#^*p* < 0.05 compared with the KLK 8 h group. The scale bar represents 40 *μ*m.

**Figure 3:**
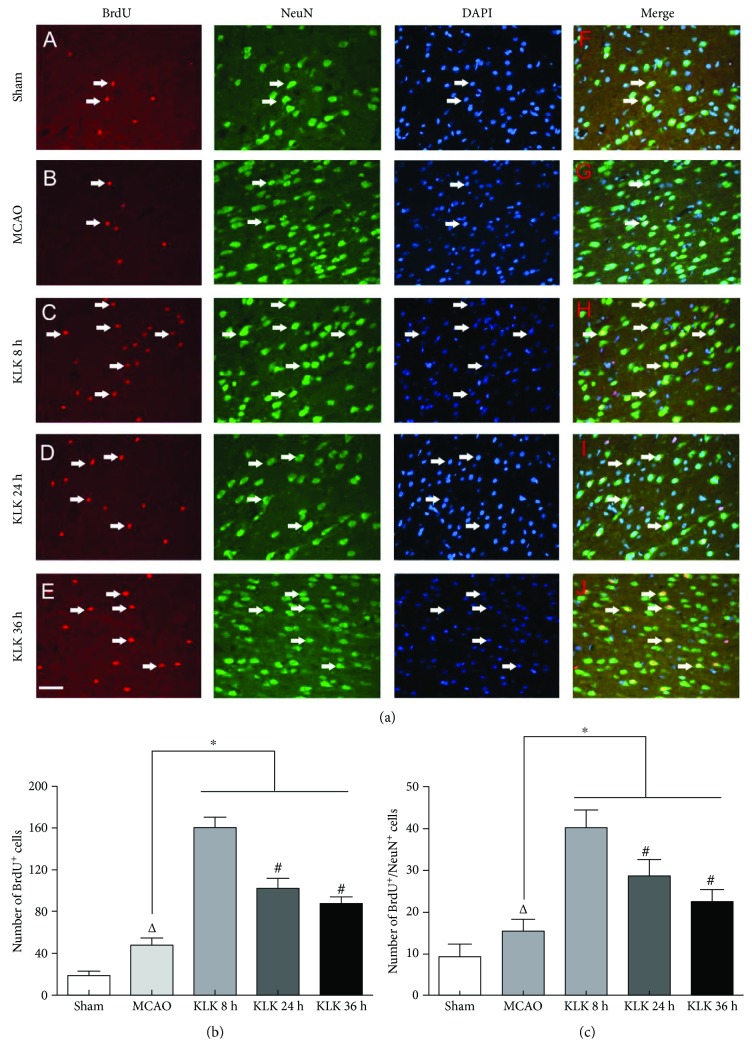
Administration of exogenous tissue kallikrein starting at 8 h, 24 h, and 36 h after ischemic stroke can increase BrdU^+^ cells (a, C–E) and BrdU^+^/NeuN^+^cells (a, H–J) in the peri-infarction region. Quantitative data of BrdU^+^cells (b) and BrdU^+^/NeuN^+^cells (c) in the peri-infarction region. ^∗^*p* < 0.05 compared with the MCAO group. ^△^*p* < 0.05 compared with the sham group. ^#^*p* < 0.05 compared with the KLK 8 h group. The scale bar represents 40 *μ*m.

Several studies have reported delayed kallikrein treatment after ischemia enhances neurogenesis and angiogenesis, which are involved in the improvement of neurological function. We next asked whether the long-term outcome difference among the three kallikrein therapeutic regimens are associated with neurogenesis. Using BrdU to label proliferative cells, we found the numbers of BrdU-positive cells in the ipsilateral SVZ and peri-infarction of ischemic animals were higher than those in the sham group (Figures 2 and 3). Compared with the MCAO group, the administration of exogenous tissue kallikrein starting at 8 h, 24 h, and 36 h after ischemic stroke can promote cell proliferation in the ipsilateral SVZ (Figures 2(a, C–E) and 2(b)) and in the peri-infarction area (Figures 3(a, C–E) and 3(b)). More importantly, we also found that there were more BrdU-positive cells in the KLK 8 h group than in the KLK 24 h and KLK 36 h groups both in the ipsilateral SVZ and in the peri-infarction area (Figures 2(b) and 3(b)). However, there was no significant difference between the KLK 24 h and KLK 36 h groups (in the ipsilateral SVZ, 127.17 ± 12.54 versus 131.67 ± 9.58, *p* > 0.05; in the peri-infarction area, 99.33 ± 9.93 versus 85.5 ± 4.04, *p* > 0.05). The results suggest that more cell proliferation after focal cerebral infarction in the KLK 8 h group might be associated with its better neurological efficacy.

### 3.3. Kallikrein Treatment Starting at 8 h after Ischemia Induces More Neural Stem Cell Proliferation

Nestin, an intermediate filament protein, is a marker of a neuroepithelial stem cell. As Nestin is a marker of neuroepithelial stem cell, we used double immunolabeling of Nestin and BrdU to show the proliferation of neural stem cells. All kallikrein treatment regimens significantly elevated the number of BrdU^+^/Nestin^+^ cells in the ipsilateral SVZ compared with the MCAO group (Figures 2(a, H–J) and 2(c), all *p* < 0.05). The more neural stem cells were observed in the KLK 8 h group than in the KLK 24 h and KLK 36 h groups (Figure 2(c), *p* < 0.05). However, there was no significant difference between the KLK 24 h group and KLK 36 h group (47.83 ± 3.76 versus 44.83 ± 2.71, *p* > 0.05).

### 3.4. Kallikrein Treatment Starting at 8 h after Ischemia Enhances More Differentiation of Neural Stem Cell into Mature Neuron

**Figure 4:**
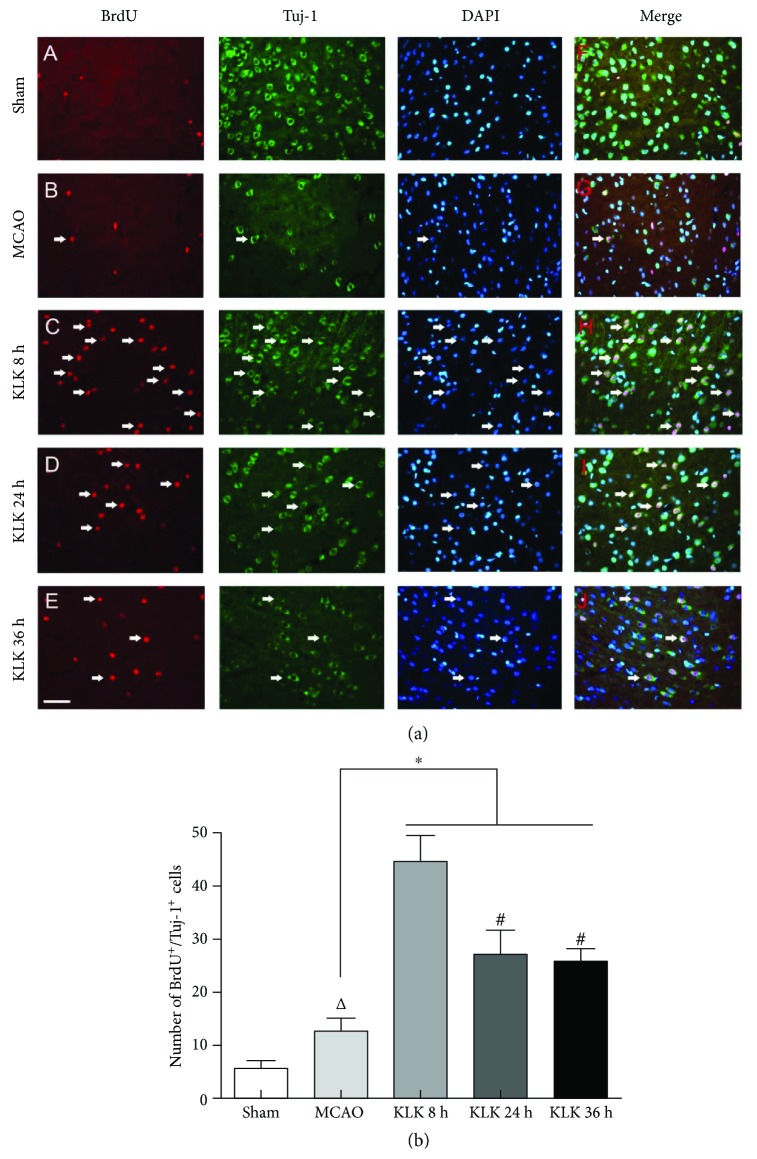
Administration of exogenous tissue kallikrein starting at 8 h, 24 h, and 36 h after ischemic stroke can increase BrdU^+^ cells (a, C–E) and BrdU^+^/Tuj-1^+^ cells (a, H–J) in the peri-infarction region. Quantitative data of BrdU^+^/Tuj-1^+^cells (b) in the peri-infarction region. ^∗^*p* < 0.05 compared with MCAO group. ^△^*p* < 0.05 compared with the sham group. ^#^*p* < 0.05 compared with the KLK 8 h group. The scale bar represents 40 *μ*m.

Next, we used NeuN and Tuj-1, two markers of mature neurons, to check whether kallikrein treatment can induce mature neuron differentiated from proliferative neural stem cells. Compared with the MCAO group, the therapy of exogenous tissue kallikrein did augment both BrdU^+^/NeuN^+^ cells (Figures 3(a, H–J) and 3(c), all *p* < 0.05) and BrdU^+^/Tuj-1^+^ cells (Figures 4(a, H–J) and 4(b), all *p* < 0.05) in the peri-infarction region. Negative control staining of NeuN or Tuj-1 was shown in the supplementary material (available here). The BrdU^+^/NeuN^+^ cells and BrdU^+^/Tuj-1^+^ cells appeared more in the KLK 8 h group than in the KLK 24 h and KLK 36 h groups (Figure 3(c), all *p* < 0.05; Figure 4(b), all *p* < 0.05). Nevertheless, there was no significant difference between the KLK 24 h group and KLK 36 h group (BrdU^+^/NeuN^+^ cells, 27.17 ± 2.32 versus 25.17 ± 2.41, *p* > 0.05; BrdU^+^/Tuj-1^+^ cells, 27.67 ± 2.8 versus 25.33 ± 2.25, *p* > 0.05).

### 3.5. Kallikrein Treatment Starting at 8 h after Ischemia Promotes More Angiogenesis in Peri-Infarct Area

**Figure 5:**
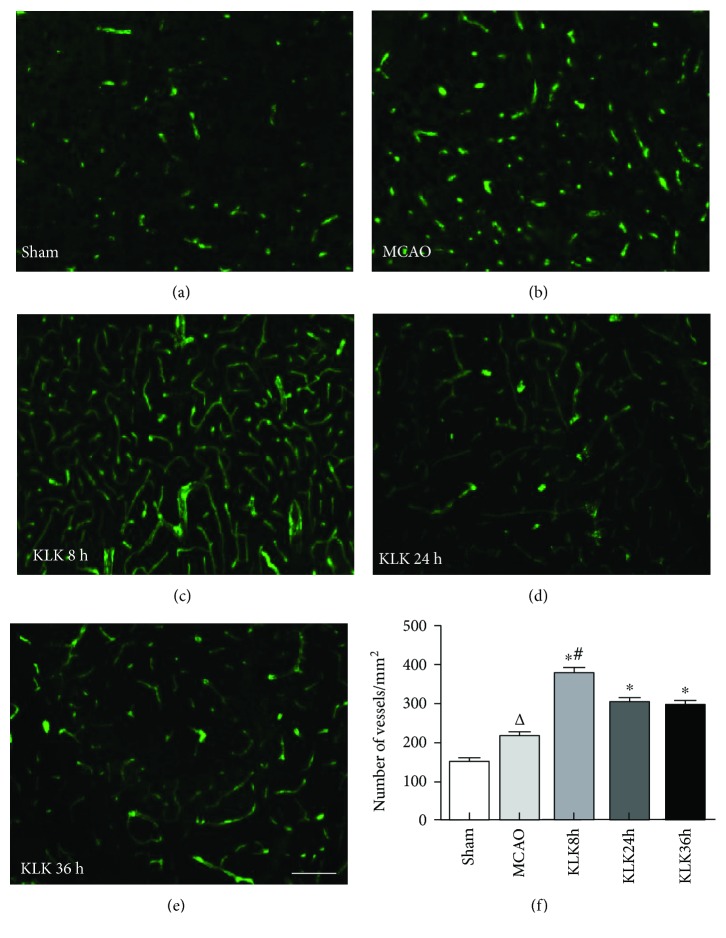
Administration of exogenous tissue kallikrein starting at 8 h, 24 h, and 36 h after ischemic stroke can increase vWF^+^ cells (c–e) compared with the MCAO group in the peri-infarction region. (f) Quantitative data of vWF^+^ cells in the peri-infarction region (Mean ± SD). ^∗^*p* < 0.05 compared with the MCAO group. ^△^*p* < 0.05 compared with the sham group. ^#^*p* < 0.05 compared with the KLK 24 h group and KLK 36 h group. The scale bar represents 50 *μ*m.

Angiogenesis also plays an important role in the long-term outcome after ischemia. In this study, the angiogenesis in the peri-infarction region was analyzed by immunostaining with Von Willebrand factor (vWF), an endothelial cell marker. Compared with the MCAO group, three kallikrein therapeutic regimens significantly increased the expression of markers of vessel in the peri-infarction region (Figures 5(c)–5(f), all *p* < 0.05), indicating kallikrein may have stimulated endothelial cell proliferation and promoted the new vessel formation. Among three kellikrein treatment groups, the KLK 8 h group had more new vessel than the KLK 8 h and KLK 36 h groups (Figure 5(f), *p* < 0.05). However, there was no significant difference between the KLK 24 h group and KLK 36 h group (301 ± 21.09 versus 295 ± 23.7, *p* > 0.05).

### 3.6. Delayed Kallikrein Treatment Promotes Angiogenesis through AKT-GSK3*β*-VEGF Signaling Pathway

**Figure 6:**
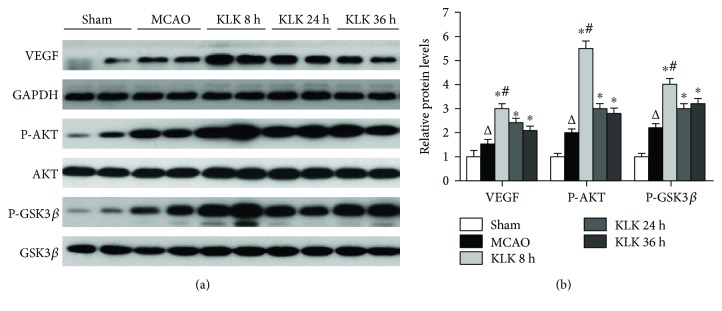
Administration of kallikrein starting at 8 h, 24 h, and 36 h after ischemic stroke activated AKT-GSK3*β*-VEGF signaling pathway. (a) Western blots of ipsilateral cerebrocortical homogenates from mice at 14 days after ischemia. (b) The blots were quantified, and the relative levels of VEGF, P-AKT, and P-GSK3*β* (Mean ± SD) after normalization with the GAPDH, AKT, and GSK3*β*, respectively. ^∗^*p* < 0.05 compared with the MCAO group; ^△^*p* < 0.05 compared with the sham group; ^#^*p* < 0.05 compared with the KLK 24 h group and KLK 36 h group.

Several studies had reported that inhibiting inflammation, oxidative stress, and increasing NO and VEGF formation are in angiogenesis enhanced by kallikrein. To figure out the molecular mechanisms, by which the kallikrein treatment starting at 8 h after ischemia has more angiogenesis, we explored the AKT-GSK3*β*-VEGF signaling pathway after kallikrein treatment. The results show that kallikrein treatment significantly promoted VEGF expression at peri-infarct zone compared with the MCAO group (Figures 6(a) and 6(b)). Consistent with the angiogenesis change tendency in different kallikrein treatment groups, KLK 8 h have more VEGF expression level than the other two treatment groups. Furthermore, we found that the upstream signal molecules in AKT-GSK3*β*-VEGF pathway were also activated after kallikrein treatment, that is, increased AKT (Ser473) and GSK3*β* (Ser9) phosphorylation, and thus GSK3*β* activity was reduced. The data suggest that delayed kallikrein treatments can activate AKT-GSK3*β*-VEGF signaling pathway, which is involved in agiogenesis enhancement. More importantly, the better effect of kallikrein treatment at 8 h after ischemia was associated with more activation of AKT-GSK3*β*-VEGF pathway.

## 4. Discussion

Previous studies have showed delayed kallikrein gene delivery or protein infusion at 8 h, 24 h, or less than 48 h after ischemia was effective in reducing neurological deficit [10, 15]. However, it is still unclear of which time point for the delayed kallikrein treatment will produce better protective effect. In this study, we confirmed that delayed administration of exogenous kallikrein protein starting at 8 h, 24 h, and 36 h after ischemic stroke onset protects against neurological dysfunction. More importantly, kallikrein treatment starting at 8 h after ischemia rendered better long-term outcome than kallikrein treatment starting at 24 h and 36 h. Besides, the molecular mechanisms underlying the protection of delayed kallikrein treatment are associated with neurogenesis and angiogenesis.

In this study, two neurological function tests were applied to evaluate the effect of the delayed kallikrein on the neurological recovery at 14 days and 28 days after ischemia. The first one is modified neurological severity score (mNSS) including motor, sensory, balance, and reflex tests. Another is pole test, which can further assess the motor function. Although the delayed kallikrein treatment starting at 8 h, 24 h, and 36 h all can reduce the mNSS and floor time and turn time, which was consistent with the previous study, the treatment starting at 8 h had better neurological recovery than the other two time points. Furthermore, we found kallikrein at 8 h also significantly reduced brain loss compared with the treatment at 24 h and 36 h. It is interesting that kallikrein treatment starting at 36 h after stroke only reduced neurological deficit and did not decrease brain loss. It may be explained that the cerebral infarct lesions were formed and irreversible within 36 h after stroke onset. Indeed, several therapeutic agents treating ischemic stroke can ameliorate neurological function instead of reducing infarction volume [16–18].

After comparing the effect of a delayed kallikrein treatment starting at different time points, we next explored the mechanisms accounting for the protection difference among three kallikrein therapeutic regimens. Several molecular mechanisms are associated with kallikrein-enhanced protection, such as inhibiting apoptosis and inflammation and reducing oxidative stress. The neurogenesis and angionesis play a critical role in the long-term neurological recovery. For the neurogenesis mechanism, we used the BrdU, a thymidine analog, to label proliferative cells. The BrdU-positive cells in the SVZ and the peri-infarction cortex were induced by cerebral ischemia. Delayed kallikrein treatment starting at 8 h, 24 h, and 36 h significantly raised the number of BrdU-positive cells compared with the MCAO group suggesting that delayed kallikrein protection against stroke was associated with cell proliferation enhancement. Paralleling with the increase of BrdU-labeled cells, BrdU/Nestin^−^-positive cells were also increased in the SVZ in all delayed kallikrein treatment groups, which suggested that delayed kallikrein treatment also promotes endogenous neural stem cell (NSCs) proliferation. NSCs will play a role after differentiation into mature neurons.

By the use of colabeling of BrdU with a mature neuronal marker NeuN or Tuj-1, we found that exogenous kallikrein treatment starting at 8 h, 24 h, or 36 h significantly elevated the number of BrdU^+^/NeuN^+^ and BrdU^+^/Tuj-1^+^ cells in the peri-infarction cortex. These data indicate that delayed treatment can enhance proliferation and differentiation of NSCs into neuron. Although delayed kallikrein treatment at all time points can promote the neurogenesis, more importantly, kallikrein treatment starting at 8 h after ischemia increased more NSC cells in the SVZ and mature neurons in peri-infarction region than the other two time point treatments. These results suggest that more neurogenesis for kallikrein treatment starting at 8 h after ischemia might contribute to its better long-term outcome.

Postischemic angiogenesis also plays an important role in the long-term functional recovery [19]. Previous studies have shown that kallikrein promotes angiogenesis in diverse peripheral tissue, such as hindlimb ischemia, cardiac infarction, and renal ischemia [20, 21]. In this study, we found that, compared with the MCAO group, delayed treatment with kallikrein starting at all time points significantly increased vascular density at 14 days after ischemia in peri-infarction area, suggesting that delayed kallikrein treatment can promote angiogenesis of brain ischemia. These results are consistent with the previous reports. Similar to neurogenesis, kallikrein treatment at 8 h can induce more angiogenesis than that at 24 h and 36 h. Kallikrein gene has been documented that it can promote neovascularization in limb ischemia and myocardial infarction [20, 22–24]. Kallikrein has also been proven to stimulate angiogenesis after local brain infarction [10, 15]. Our data demonstrate that the beneficial effects of delayed kallikrein treatment are exerted via enhanced angiogenesis in the peri-infarction zone, and the distinct therapy effect of kallikein at different times after stroke is in accordance with the degree of vascular proliferation.

PI3K-AKT activation plays an important role in angiogenesis by regulating VEGF expression [25]. VEGF is a well-known endothelial cell-specific angiogenic factor that regulates angiogenesis through the stimulation of proteolytic activity as well as endothelial cell proliferation and migration [26].

Indeed, previous reports showed kallikrein gene transfer protected against acute phase myocardial infarction by promoting neovascularization and improving cardiac function by increasing AKT and GSK3*β* phosphorylation and thus reducing GSK3*β* activity. In this study, we found all delayed kallikrein protein treatment significantly upregulated VEGF expression in peri-infarct area, paralleling to AKT and GSK3*β* phosphorylation. Notably, delayed kallikrein treatment at 8 h induced more activation of this pathway than kallikrein treatment starting at other two time points. These results suggest that delayed kallikrein protein treatment can activate the AKT-GSK3*β*-VEGF pathway, which might be associated with vascular proliferation enhancement.

In this study, we showed that kallikrein starting at 8 h after ischemic onset is more effective than that at 24 h and 36 h. the results suggested that, for delayed kallikrein administration, earlier injection is better. But, to determine appropriate delivery time, further research should be done by comparing delayed treatment with early treatment (e.g., 4 h or earlier).

In summary, our research show that although delayed systemic delivery of exogenous kallikrein starting at 8 h, 24 h, and 36 h provides protection against cerebral infarction, kallikrein treatment starting at 8 h after ischemia has better long-term outcome than kallikrein starting at 24 h and 36 h. Besides, the long-term outcome difference of exogenous kallikrein treatment starting at three time points is closely associated with the degree of neurogenesis and angiogenesis. These findings will contribute to choose the appropriate time for better clinical application of exogenous kallikrein.

## Supplementary Materials

Supplementary Materials Negative control for Figure 3 and Figure 4.
