# Supplementary figures and images for: The Long-Term Outcome Comparison of Different Time-Delayed Kallikrein Treatments in a Mouse Cerebral Ischemic Model
Source: Stem Cells Int. 2018 Apr 5;2018:1706982. doi: 10.1155/2018/1706982 (PMC5907522; doi:10.1155/2018/1706982)

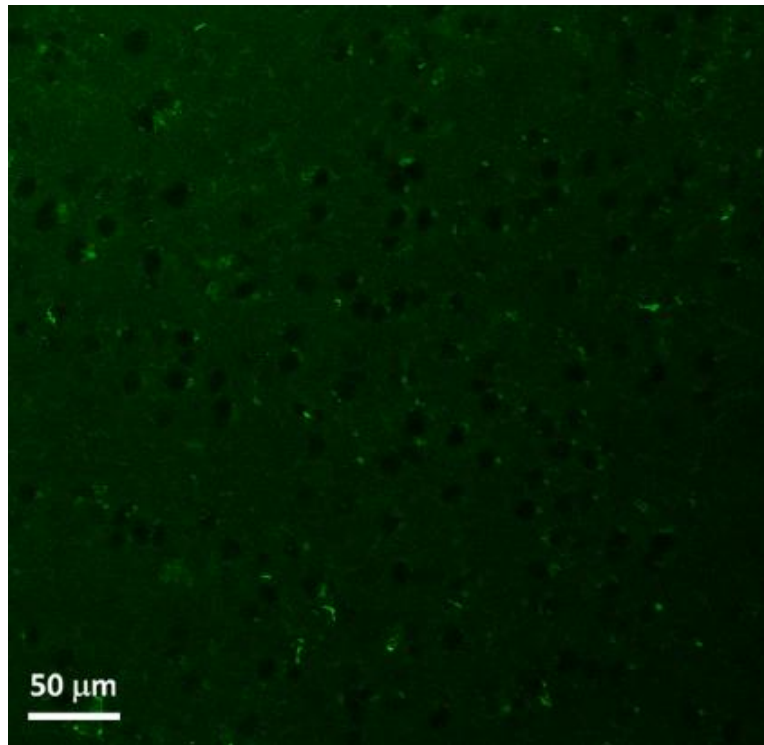

Negative control for NeuN in Fig.3

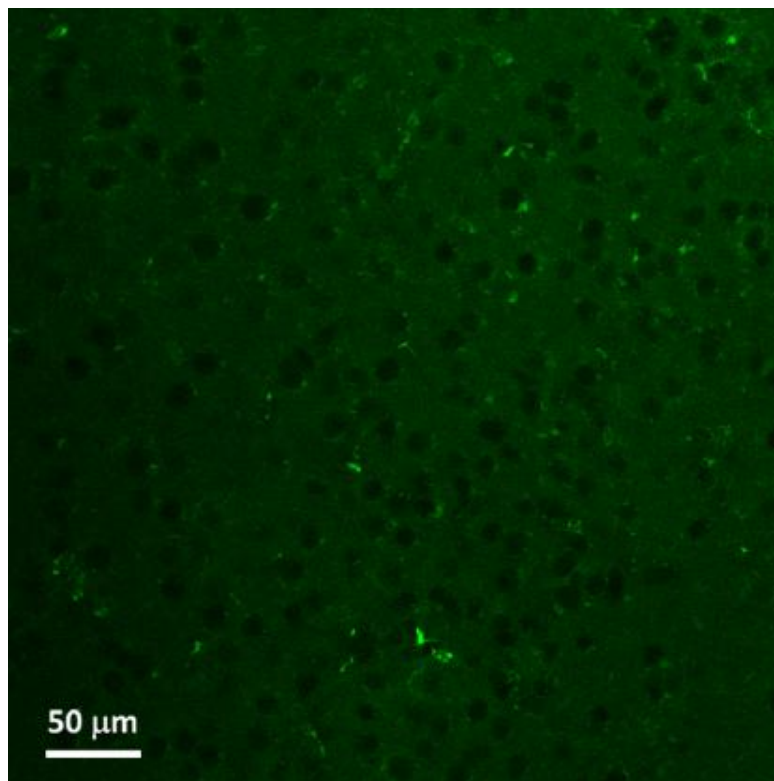

Negative control for Tuj-1 in Fig.4

Supplement: Supplementary Materials — Negative control for Figure 3 and Figure 4. [file 1706982.f1.pdf]
